# A Weakened Transcriptional Enhancer Yields Variegated Gene Expression

**DOI:** 10.1371/journal.pone.0000033

**Published:** 2006-12-20

**Authors:** Cathy Collins, Peter Azmi, Maribel Berru, Xiaofu Zhu, Marc J. Shulman

**Affiliations:** Department of Immunology, University of Toronto Toronto, Canada; University of California, San Diego, United States of America

## Abstract

Identical genes in the same cellular environment are sometimes expressed differently. In some cases, including the immunoglobulin heavy chain (IgH) locus, this type of differential gene expression has been related to the absence of a transcriptional enhancer. To gain additional information on the role of the IgH enhancer, we examined expression driven by enhancers that were merely weakened, rather than fully deleted, using both mutations and insulators to impair enhancer activity. For this purpose we used a LoxP/Cre system to place a reporter gene at the same genomic site of a stable cell line. Whereas expression of the reporter gene was uniformly high in the presence of the normal, uninsulated enhancer and undetectable in its absence, weakened enhancers yielded *variegated* expression of the reporter gene; i.e., the average level of expression of the same gene differed in different clones, and expression varied significantly among cells within individual clones. These results indicate that the weakened enhancer allows the reporter gene to exist in at least two states. Subtle aspects of the variegation suggest that the IgH enhancer decreases the average duration (half-life) of the silent state. This analysis has also tested the conventional wisdom that enhancer activity is independent of distance and orientation. Thus, our analysis of mutant (truncated) forms of the IgH enhancer revealed that the 250 bp core enhancer was active in its normal position, ∼1.4 kb 3′ of the promoter, but inactive ∼6 kb 3′, indicating that the activity of the core enhancer was distance-dependent. A longer segment – the core enhancer plus ∼1 kb of 3′ flanking material, including the 3′ matrix attachment region – was active, and the activity of this longer segment was orientation-dependent. Our data suggest that this 3′ flank includes binding sites for at least two activators.

## Introduction

Single cell assays of gene expression in metazoa have revealed that the same gene in the same cellular environment can sometimes be expressed in more than one way [1], [2]. That is, gene expression is only partly determined by the available transcription factors and nucleotide sequences, and this indeterminism contributes importantly to ontogenetic differentiation. In extreme cases, *viz*., imprinted genes and the female mammalian X chromosome, the two alleles are differentially expressed in nearly all cells throughout the life of the organism. Some tissue-specific genes are also monoallelically expressed. For example, in the case of the odorant receptors, which comprise a large family of genes, only one allele is expressed in an individual olfactory neuron [3]. Another particularly interesting case is the interleukin 4 (IL4) gene, which is usually expressed in activated T cells: individual clones each express only one IL4 allele, and that allele is expressed at a characteristic and different rate in different clones [4]. These phenomena raise several questions: what is the epigenetic mark that determines that two otherwise identical alleles are differentially expressed, how is this mark inherited through multiple cell divisions, and what feature renders a gene susceptible to this form of differential expression?

Some evidence suggests that the occurrence of multiple states of expression is in some way related to the action of transcriptional enhancers (reviewed in [5]). Thus, genes that have been manipulated to lack an enhancer can sometimes exist in at least two states of expression for many weeks. In one such case involving the endogenous immunoglobulin heavy chain (IgH) locus, removal of the enhancer from the IgH locus of a B cell line creates conditions in which the heavy chain gene can exist in different states of expression, ranging from highly expressed to silent [6]–[8]. The different states are maintained through many cell divisions by a *cis*-acting mechanism. Cells switch from one state to another, but switching is sufficiently infrequent that distinct types of clones are recognizable, and subclones generally, but not always, resemble the parent clone. Thus, these different states are metastable, and heavy chain expression in each clone is typically variegated.

Because the endogenous IgH locus is beset with redundant regulatory elements, it is difficult to identify the direct effects of deleting the enhancer. We sought therefore to generate variegated expression with simple transgenes and then to identify molecular requirements for variegated expression. As described here, we measured expression not only in the presence and absence of the IgH enhancer, but also in intermediate circumstances, using weakened enhancers. In particular, we examined expression of a reporter gene that was linked to an enhancer that had been weakened either by partial deletions or by inserting a leaky insulator. Whereas expression was uniformly high in the presence of the enhancer and undetectable in its absence, expression driven by the weakened enhancers was variegated. That is, in the case of weakened enhancers, expression of the reporter gene was maintained at different levels in different clones and varied significantly among cells within individual clones. These results lead us to conclude that genes in which expression is driven by a weak enhancer can persist in more than one state of expression for many cell divisions. Subtle features of this variegation suggest that the function of the enhancer is to decrease the average duration (half-life) of the silent state rather than to increase the duration of the expressed state.

In the course of this analysis we have also obtained results that activity of the IgH enhancer depends strongly on the distance and orientation to the promoter.

## Results

### Structure and assay of the reporter gene

**Figure 1:**
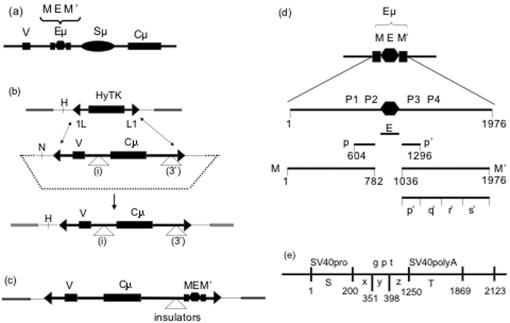
Structure of the reporter cassettes a) The endogenous μ gene. The boxes labeled V and C represent the exons encoding the variable (V) and constant (Cμ) regions of the immunoglobulin μ heavy chain gene. The relative positions of the intronic enhancer (Eμ) and switch (Sμ) regions in the V-C intron are shown. The Eμ enhancer is depicted with three components: the core enhancer (E) flanked by matrix attachment regions, M and M′. b) Recombination-mediated cassette exchange. The upper panel depicts a DNA segment in which (inverted) LoxP sites (1L and L1) flank a gene encoding the HyTK fusion protein (hygromycin-resistance and thymidine kinase [gancyclovir sensitivity]). As described previously, this DNA segment was inserted in the genome of the recipient hybridoma cell line [9]. The HyTK and μ cassettes are represented as thick lines, with major exons as rectangles, the LoxP sites as triangles (L1 in the “forward” orientation, 1L in the “reverse” orientation). The three-stranded line represents the chromosomal DNA. The residual backbone of the vector that is shared between the target and the replacement is represented as a thin line; the remainder of the reporter cassette is represented as a dotted line. The middle panel depicts the structure of the replacement vector designed to substitute a modified μ gene for the Hy-TK gene via Cre-mediated recombination of the LoxP sites. This vector lacks the switch (Sμ) region and was constructed by joining two segments of the μ gene of the Sp6 hybridoma: The 2.0 kb V-bearing segment includes the DNA between the PacI site (∼850 bp 5′ of the initiator ATG) and the NgoMIV site 3′ of J4. The 4.6 kb Cμ-bearing segment includes the DNA between the SnaI site 5′ of Cμ and the SphI site 3′ of Cμ. DNA segments were inserted either in the intron at the NgoMIV site (denoted i), 1.2 kb 3′ of the initiator ATG, or 3′ of Cμ at the SphI site (denoted 3′), 5.9 kb 3′ of the ATG. The lower panel depicts the structure after the μ reporter cassette has replaced the HyTK gene. To distinguish replacements from random insertions we made use of the HinDIII (H) and NheI (N) sites that distinguish the DNA that flanks the HyTK and μ genes. The notations (i) and (3′) indicate the two sites where enhancer-derived segments were inserted. c) Structure of the reporter gene used for assaying insulator segments. d) The enhancer-derived segments. The “full” enhancer corresponds to the 2034 bp DNA segment bounded by the NgoMIV and Bst1107I sites, which are denoted as nucleotides 1 and 2034, respectively. The indicated subsegments were prepared by PCR, and nucleotide positions of their endpoints, numbered from the first nucleotide of the NgoMIV site are as follows: M, 1-782; E, 783-1035; M′, 1036-2034; p, 604-782; p′, 1036-1295; q′, 1296-1342; r′, 1343-1654; s′, 1655-1976. The XbaI sites that are often used to delimit the MARs are at 448 and 1441. The Bright binding sites are P1, 624-648; P2, 733-767; P4 1183-1202; P4, 1237-1276. e) Subsegments of the *gpt* cassette. The full *gpt* expression cassette includes the SV40 promoter (S), the *gpt* structural gene (gpt) and the SV40 polyA site (T). The *gpt* structural gene was divided into three subsegments, denoted x, y z. The nucleotide positions are measured from the first nucleotide of the SphI site in the SV40 promoter. The figures are not to scale.

In order to measure expression of reporter genes that are all in the same genomic context, we used recombination-mediated cassette exchange (RMCE) to construct isogenic cell lines in which a single transfected reporter gene was inserted at a reproducible genomic site in a hybridoma cell line. The RMCE system, which uses Cre recombinase and the cognate LoxP sites, is illustrated in Figure 1 and is described in detail elsewhere [9]. The reporter gene and recipient cell line are derived from the mouse hybridoma cell line, Sp6, which secretes IgM(κ) specific for the hapten trinitrophenyl. The reporter gene is a truncated version of the Sp6 immunoglobulin μ heavy chain gene, and the recipient cell line, Z10/HyTK, also derived from Sp6, lacks the endogenous μ gene but still expresses the κ light chain. Transfectants of Z10/HyTK that express the μ gene therefore incorporate the μ protein into IgM, which is then secreted into the culture medium. The recipient cell line bears a single copy of the “target” HyTK fusion gene, which confers resistance to hygromycin and sensitivity to gancyclovir and which is flanked by LoxP sites. Replacement of the HyTK target with the μ reporter gene renders the cells gancyclovir-resistant (gan^R^). The LoxP sites (denoted L1 and 1L) are inverted with regard to each other in order to prevent Cre-mediated excision of the target cassette [10]. In previous work we analyzed numerous independent replacements bearing the reporter gene in one particular (reversed) orientation [9]. In this chromosomal site and in this orientation expression of the reporter gene is uniform and reproducible in the presence of the IgH enhancer, and no expression is detectable in the absence of an enhancer. These features suggested that this system might be suitable for identifying conditions for variegated expression.

The endogenous μ gene includes an enhancer in the second (J-Cμ) intron, and our original μ reporter gene had this same arrangement. Because altering the enhancer in this position might alter the processing of this transcript and thus obfuscate effects on transcription, the enhancer and insulator were placed 3′ of the transcription unit (Fig. 1). Thus, the reporter cassettes had one of three general structures: (a) the enhancer in the J-Cμ intron, (b) the enhancer 3′ of Cμ and (c) an insulator inserted between Cμ and the enhancer in the 3′ position.

As noted above, previous work on the endogenous IgH locus showed that deletion of the intronic enhancer resulted in variegated expression of the μ gene [6]–[8]. Variegated expression in the endogenous locus of the hybridoma cells was evident in two ways: clonal heterogeneity (differences among clones in their average level of expression) and cellular heterogeneity (differences in expression among cells within a clone). In order to discern in the simpler RMCE system whether the structure of the reporter gene uniquely determined expression or allowed variegation, we examined μ expression by multiple independent replacements with the same reporter gene (Table 1). As the first step, we selected gan^R^ transfectants, which were then tested with a simple PCR-based assay to identify colonies (replacements) in which the reporter cassette had replaced the target cassette in the reversed orientation [9]. As an initial test for variegation, the culture fluid of each replacement was assayed for secreted IgM by ELISA, and those replacements with the lowest (denoted /a) and highest (denoted /b) titers were examined further to confirm that the μ reporter cassette was complete and that there were no additional copies of the reporter either at the target site or elsewhere in the genome. The ELISA used for this initial screening had a three-fold reproducibility. Thereafter, expression of the reporter gene was examined more precisely and informatively using both clonal (ELISA, Northern blot) and single cell (flow cytometry) assays. As presented below, in many cases there was no significant difference between independently derived transfectants. In other cases, independent transfectants expressed the same reporter gene differently and were investigated further.

**Table 1:**
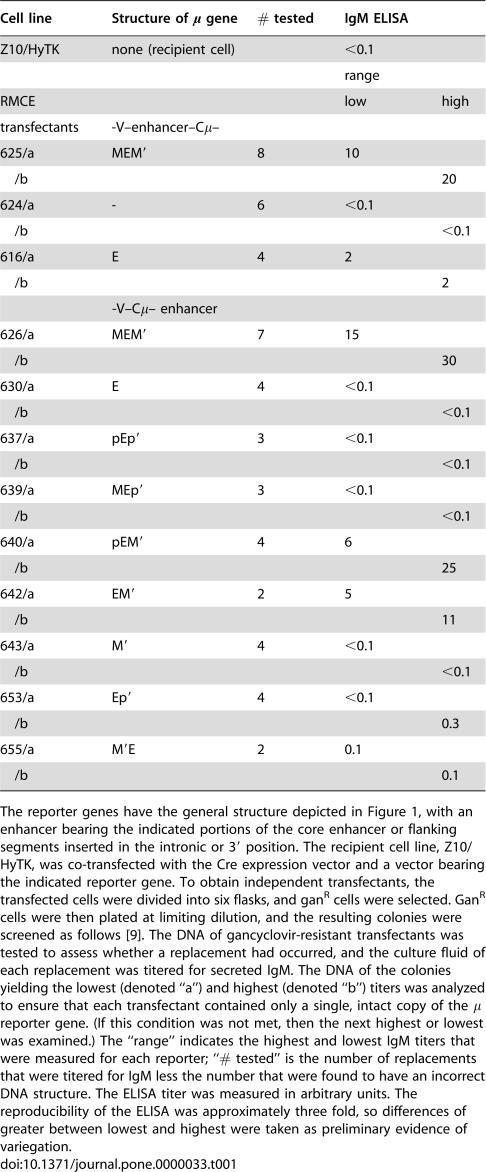
Position-dependent and orientation-dependent activity of the core enhancer.

Cell line	Structure of μ gene	# tested	IgM ELISA	
Z10/HyTK	none (recipient cell)		<0.1	
			range	
RMCE			low	high
transfectants	-V–enhancer–Cμ–			
625/a	MEM′	8	10	
/b				20
624/a	-	6	<0.1	
/b				<0.1
616/a	E	4	2	
/b				2
-V–Cμ– enhancer				
626/a	MEM′	7	15	
/b				30
630/a	E	4	<0.1	
/b				<0.1
637/a	pEp′	3	<0.1	
/b				<0.1
639/a	MEp′	3	<0.1	
/b				<0.1
640/a	pEM′	4	6	
/b				25
642/a	EM′	2	5	
/b				11
643/a	M′	4	<0.1	
/b				<0.1
653/a	Ep′	4	<0.1	
/b				0.3
655/a	M′E	2	0.1	
/b				0.1

The reporter genes have the general structure depicted in Figure 1, with an enhancer bearing the indicated portions of the core enhancer or flanking segments inserted in the intronic or 3′ position. The recipient cell line, Z10/HyTK, was co-transfected with the Cre expression vector and a vector bearing the indicated reporter gene. To obtain independent transfectants, the transfected cells were divided into six flasks, and gan^R^ cells were selected. Gan^R^ cells were then plated at limiting dilution, and the resulting colonies were screened as follows [9]. The DNA of gancyclovir-resistant transfectants was tested to assess whether a replacement had occurred, and the culture fluid of each replacement was titered for secreted IgM. The DNA of the colonies yielding the lowest (denoted “a”) and highest (denoted “b”) titers was analyzed to ensure that each transfectant contained only a single, intact copy of the μ reporter gene. (If this condition was not met, then the next highest or lowest was examined.) The “range” indicates the highest and lowest IgM titers that were measured for each reporter; “# tested” is the number of replacements that were titered for IgM less the number that were found to have an incorrect DNA structure. The ELISA titer was measured in arbitrary units. The reproducibility of the ELISA was approximately three fold, so differences of greater between lowest and highest were taken as preliminary evidence of variegation.

### Activity of the enhancer in the 3′ position

The intronic enhancer of the IgH locus includes a “core” enhancer (denoted E) that has binding sites for numerous transcription-activating factors. The sequences flanking the core bind to the nuclear matrix and are therefore referred to as “matrix attachment regions” (MARs, denoted as M and M′ in Figure 1). Other work showing that the core enhancer was sufficient to stimulate expression in transfected cell lines suggested that the core enhancer would be sufficient to stimulate expression of the μ reporter in our system. Table 1 presents the initial ELISA measurements of secreted IgM from replacements bearing different reporter genes. Within the experimental error (∼3 fold) of this ELISA, we detected no significant difference in μ expression among transfectants bearing the same reporter gene. As presented in Table 1, the core enhancer stimulated μ expression only when located in its normal intronic position (#616), 1.4 kb 3′ of the promoter, and not when it was 6 kb 3′ of the promoter (#630). By contrast, the full enhancer, i.e., the core enhancer flanked by the MARs, was active in both the intronic (#625) and 3′ positions (#626). We conclude that the activity of the core enhancer is strongly distance-dependent.

The foregoing finding suggested that the core enhancer was strengthened by an element (s) in the adjoining M or M′ segments. The segments denoted p and p′ each include two binding sites for a transcriptional activator, Bright [11], and we considered that these sites might account for difference in activity between the core and full enhancers. We therefore constructed truncated versions of MEM′ that included or lacked p or p′ and tested by ELISA for secreted IgM whether they stimulated expression (Table 1). These results indicated that most or all of the activity that differentiated MEM′ from E was included in M′ and that p′ was not sufficient. As expected from earlier analyses of the M and M′ segments [12], M′ alone was inactive.

It is commonly stated that the activity of enhancers is independent of orientation. In the light of the distance dependence of the core enhancer, we also tested the effect of inverting the EM′ segment. As indicated in Table 1, in the *inverted* orientation, M′E (#655) was *inactive*. These findings, which suggest that the activity of these forms of the enhancer is strongly sensitive to both distance and orientation, are considered further in the Discussion.

**Figure 2:**
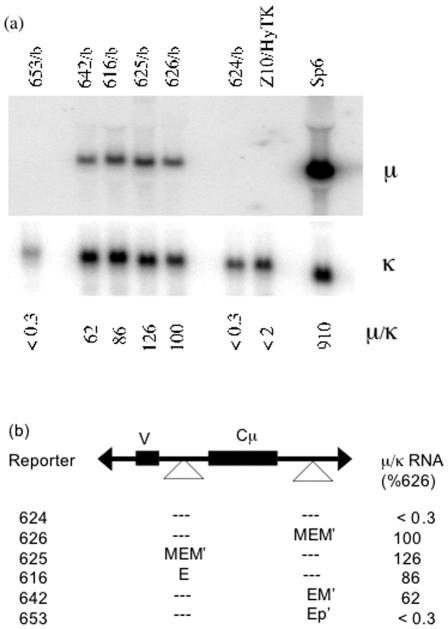
Analysis of μ expression by northern blot a) As described in the text, multiple independent replacements were isolated for each vector, and the concentration of IgM in culture supernatant of these cell lines was measured by ELISA (Table 1). Total RNA was isolated from representative cell lines and analyzed by Northern blot probed with segments of the μ and κ genes. The intensity of the bands was quantified by phosphorimager, and the μ/κ ratio, normalized to the value for cells expressing #626, is indicated below each lane. b) Results from the northern blot in (a) are listed next to a diagram of each enhancer-derived segment.

In order to assess μ expression more directly and accurately, we measured μ RNA levels by Northern blot (Fig. 2). These measurements confirmed that the core enhancer was active in the intron but not in the 3′ position, and that most of the activity associated with MEM′ was conferred by the M′ segment.

**Figure 3:**
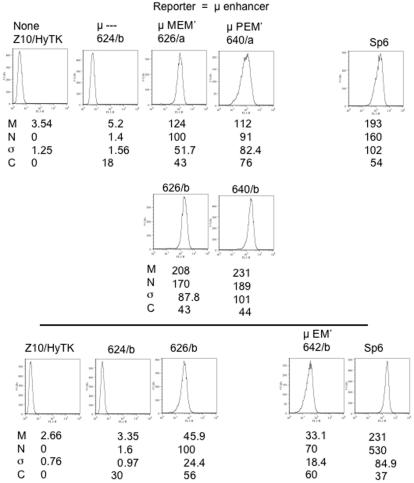
Flow cytometry of μ expression from reporter gene bearing different segments of the full enhancer. Transfectants bearing the indicated reporter genes were analyzed by flow cytometry. Cells were fixed and permeabilized, and intracellular μ chains were stained with μ-specific fluorescent antibodies. ∼10^4^ cells were then analyzed. In these histograms the horizontal axis indicates the mean fluorescence (logarithmic scale) and the vertical axis the number of cells with the corresponding fluorescence. M is the mean fluorescence for each population. Z10/HyTK, the recipient cell line was used in this case as the negative control, and its mean “fluorescence”, M_0_, for each experiment was subtracted from the mean fluorescence, M_x_, measured for a cell population expressing reporter “x”. This corrected fluorescence is compared with M_626_, the corrected fluorescence in that experiment for the reporter #626 with the full enhancer. Thus, the “normalized” fluorescence, N_x_, for reporter “x” was calculated as N_x_  =  (M_x_−M_0_)/(M_626_−M_0_), where M_626_ is the mean fluorescence for the reporter with the full enhancer and M_0_ is the fluorescence for the recipient cell line or other μ non-expresser. σ is the standard deviation of the fluorescence. In order to correct for variation in background “fluorescence”, we calculated a corrected coefficient of variation, C_x_, for reporter “x” as C_x_  =  (σ_x_^2^−σ_0_^2^)^1/2^/M_x_−M_0_, where σ_x_ and σ_0_ are the standard deviations associated, respectively, with reporter “x” and with the recipient cell line or other μ non-expresser.

The ELISA measurements of secreted IgM from independent replacements did not reveal evidence of clonal heterogeneity. i.e., independent transfectants expressing the same reporter gene secreted the same amount of IgM, within the experimental error of the IgM ELISA. To test for cellular heterogeneity, we used flow cytometry with fluorescent μ-specific antibody to measure the amount of μ protein in individual cells. The μ protein is secreted as IgM, so intracellular μ is closely related to the contemporaneous amount of μ mRNA. To quantify and compare the level of reporter gene expression we calculated a normalized mean fluorescence, N, in which mean fluorescence of different reporters was normalized to the mean fluorescence of a reporter with the full enhancer. To quantify heterogeneity, we calculated a corrected coefficient of variation, C, in which variation that was unrelated to reporter gene expression was discounted (see legend to Figure 3). These results generally indicated that expression of reporters bearing the EM′ segment, e.g., #626 and #642, was homogeneous, i.e., the value of C for these reporters was similar to that of the parental hybridoma. In the case of reporter bearing the segment pEp′ (#640), expression in replacement #640/a was reproducibly more heterogeneous than in #640/b, but we considered this effect too small to allow further analysis.

### Expression using an insulated enhancer

Analyses of imprinted genes have suggested that transcriptional insulators can exist in two states: either inactive (methylated) or active (unmethylated); the state in turn determines whether a distal enhancer can or cannot stimulate expression of a linked promoter[13], [14]. Non-physiological DNA segments can also function as insulators. In particular, our previous work on targeted recombinants of the endogenous IgH locus suggested that the *gpt* expression cassette that was used as a selectable marker includes segment with insulator activity [15]. We considered therefore that the *gpt* cassette might also assume two states and thus cause the variegated expression of the μ gene that we had previously observed in the targeted endogenous IgH locus. We confirmed using standard assays that the *gpt* cassette could act as an insulator (see Figure S1). We then constructed several replacement vectors in which segments bearing the full or truncated *gpt* cassettes were inserted 5′ of the enhancer in the reporter cassette (Fig. 1d) and examined their mode of expression.

**Figure 4:**
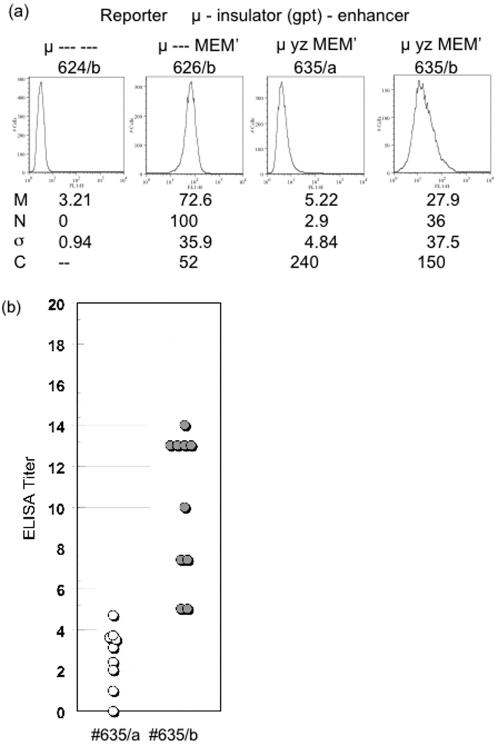
Flow cytometry of μ expression from partially insulated reporter gene. (a) Two independent transfectants expressing reporter #635 (#635/a and #635/b) were analyzed by flow cytometry, as described in Figure 3. (b) The transfectants #635/a and #635/b were subcloned, and secreted IgM was measured for each subclone. The statistical parameters, M, N, σ, and C are defined in the legend to Figure 3.

In the case of reporters bearing the complete *gpt* cassette, μ expression was undetectable in all eight independent replacements, indicating that the *gpt* cassette reduced expression by at least 80 fold, consistent with the results in the Appendix. We then sought to examine expression with a less stringent insulator and tested segments of the *gpt* cassette (Fig. 1e). Of particular interest, a segment that was wholly derived from the bacterial gene yielded variegated expression. Thus, independent transfectants, #635/a and #635/b, which bore the “yz” segment of the *gpt* structural gene, yielded heterogeneous populations that expressed the μ gene at different levels, as measured by ELISA (Table 2) and by flow cytometry (Fig. 4a). Moreover, cellular heterogeneity, as measured by the corrected coefficient of variation, C, was several fold greater for the insulated reporters than for the uninsulated enhancer (Fig. 4a). As considered further in the Discussion, cellular heterogeneity might provide insight into the role of the enhancer.

**Table 2:**
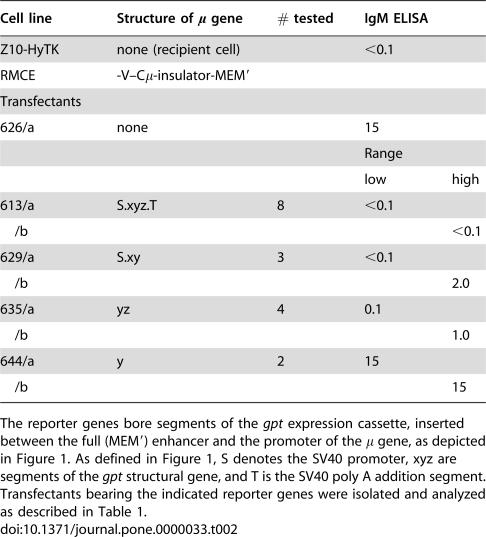
Expression from an enhancer weakened with an insulator.

Cell line	Structure of μ gene	# tested	IgM ELISA	
Z10-HyTK	none (recipient cell)		<0.1	
RMCE	-V–Cμ-insulator-MEM′			
Transfectants				
626/a	none		15	
			Range	
			low	high
613/a	S.xyz.T	8	<0.1	
/b				<0.1
629/a	S.xy	3	<0.1	
/b				2.0
635/a	yz	4	0.1	
/b				1.0
644/a	y	2	15	
/b				15

The reporter genes bore segments of the *gpt* expression cassette, inserted between the full (MEM′) enhancer and the promoter of the μ gene, as depicted in Figure 1. As defined in Figure 1, S denotes the SV40 promoter, xyz are segments of the *gpt* structural gene, and T is the SV40 poly A addition segment. Transfectants bearing the indicated reporter genes were isolated and analyzed as described in Table 1.

In the case of #635, we tested whether the distinctive level of expression was a heritable (stable) feature of these cell lines by subcloning #635/a and #635/b and measuring the level of secreted IgM by ELISA for each subclone (Fig. 4b). Each set of re-subclones resembled their parent more closely than they resembled their non-parent, thus indicating that the higher and lower expression phenotypes were sufficiently stable to be inherited in most re-subclones. Similar results were obtained for #629, which included the SV40 promoter and the “xy” segment of the bacterial *gpt* gene (data not shown).

### Expression using a mutant enhancer

**Figure 5:**
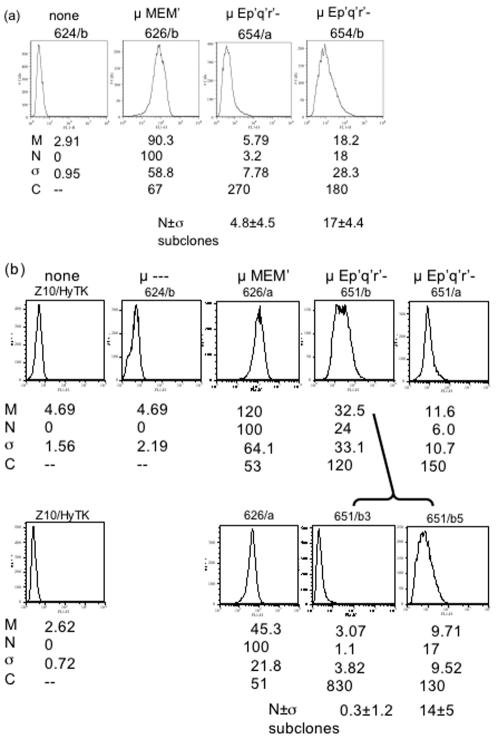
Flow cytometry of μ expression from reporters with mutant enhancers. a) Two independent transfectants expressing the #654 reporter were analyzed by flow cytometry. b) As in (a) two independent transfectants bearing reporter #651 were analyzed by flow cytometry, as described in Figure 3. The transfectant 651/b was biphasic and yielded subclones with different levels of expression, 651/b3 and 651/b5. To assess the stability of this difference, these subclones were re-subcloned, and the mean fluorescence, M, and normalized fluorescence, N, of eight re-subclones were measured. N_m_, the mean value for N for the re-subclones and the associated standard deviation, were calculated. The values of N_m_ for each set of subclones were significantly different and close to the value of the N for their respective parents. The statistical parameters, M, N, σ, and C are defined in the legend to Figure 3.

The *gpt*-derived segments that yielded variegated expression were, of course, non-physiological. Nevertheless, these results raised the question whether the variegated expression resulted from the presence of the *gpt*-derived insulator or from the weakened enhancer. Our finding that the core enhancer was strengthened by the adjoining M′ DNA suggested that some intermediate structure between EM′ and E might serve as a weakened enhancer. We therefore tested whether shortened versions of the M′ segment were active when adjoined to E (Table 3). For this purpose, the M′ segment was divided into four subregions, denoted p′q′r′s′ (Fig. 1), and truncated versions of M′ bearing different subregions were tested for activity. Each of the truncations eliminated most or all of the activity. For those truncations that allowed low but detectable expression of the μ gene, , *e.g*., enhancer segments Ep′q′r′ (#651) and E-q′r′s′ (#654), the gene structure did not uniquely determine the level of μ expression. Thus, colonies #654/a and #654/b differed in μ expression by six fold, both as measured with the ELISA for secreted IgM (Table 2), and as measured with flow cytometry of intracellular μ chain (Fig. 5). Similarly, #651/a and #651/b differed in μ expression by six fold in the ELISA and by four fold in average fluorescence. Moreover, the population of #651a was biphasic, and this colony yielded subclones, exemplified by #651/b3 and #651/b5, which differed in μ expression by ∼15 fold. To test whether lower and higher expression were heritable features, these two subclones were re-subcloned, and 8–10 re-subclones were analyzed by flow cytometry. Using mean fluorescence as a measure of μ expression, each set of re-subclones resembled their parent more closely than they resembled their non-parent, thus indicating that the higher and lower expression phenotypes were sufficiently stable to be inherited in most re-subclones. Flow cytometry indicated further that μ expression from these truncated enhancers was also associated with increased cellular heterogeneity, i.e., the corrected coefficient of variation was much larger for the mutant than for the unimpaired enhancer. In summary, reporter genes that were linked to weakened enhancers yielded variegated expression, both in the sense that independent clones often differed in their average level of μ expression, and in the sense that μ expression was heterogeneous among the cells within a single clone.

**Table 3:**
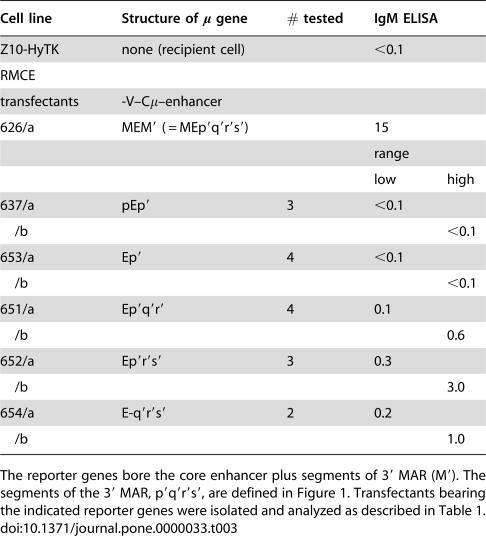
Expression from an enhancer weakened by mutation.

Cell line	Structure of μ gene	# tested	IgM ELISA	
Z10-HyTK	none (recipient cell)		<0.1	
RMCE				
transfectants	-V–Cμ–enhancer			
626/a	MEM′ ( = MEp′q′r′s′)		15	
			range	
			low	high
637/a	pEp′	3	<0.1	
/b				<0.1
653/a	Ep′	4	<0.1	
/b				<0.1
651/a	Ep′q′r′	4	0.1	
/b				0.6
652/a	Ep′r′s′	3	0.3	
/b				3.0
654/a	E-q′r′s′	2	0.2	
/b				1.0

The reporter genes bore the core enhancer plus segments of 3′ MAR (M′). The segments of the 3′ MAR, p′q′r′s′, are defined in Figure 1. Transfectants bearing the indicated reporter genes were isolated and analyzed as described in Table 1.

### Effect of 3-aminobenzamide

**Figure 6:**
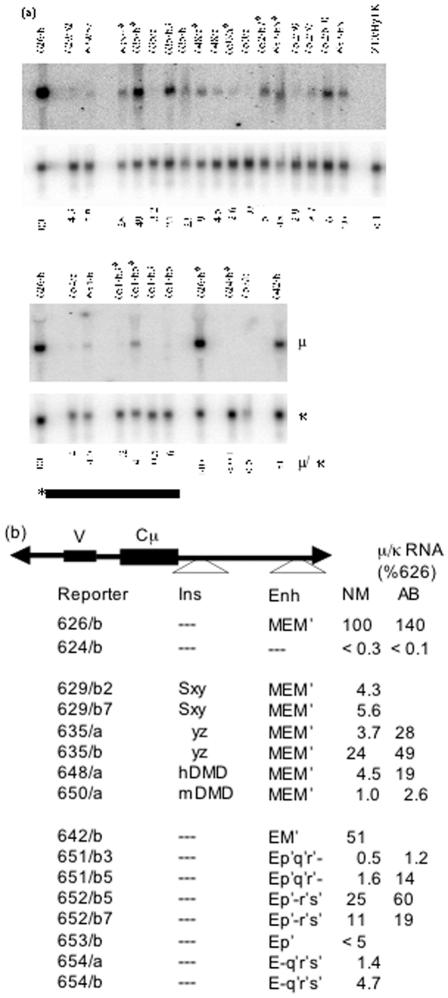
Analysis of μ RNA produced by weakened enhancers. a) Total RNA was isolated from the indicated cells and analyzed by northern blot as described in Figure 2. The asterisk indicates that the cells were incubated for approximately five days with 6.7 mM 3-aminobenzamide prior to isolating RNA. The upper panels present results for various insulators derived from the *gpt* cassette and from the igf2/H19 locus; the lower panels present results for various mutant enhancers. For vectors #629 and $635, the segments of the *gpt* cassette, S, x, y, z are defined in Figure 1. Reporters #648 and #650 bear the insulator (DMD) from the igf2/H19 loci of mouse and human, respectively. b) The normalized μ/κ ratios from (a) for cells grown in normal medium (NM) or medium supplemented with 3-aminobenzamide (AB) are listed next to diagrams showing the insulators and enhancers in the reporter genes.

**Figure 7:**
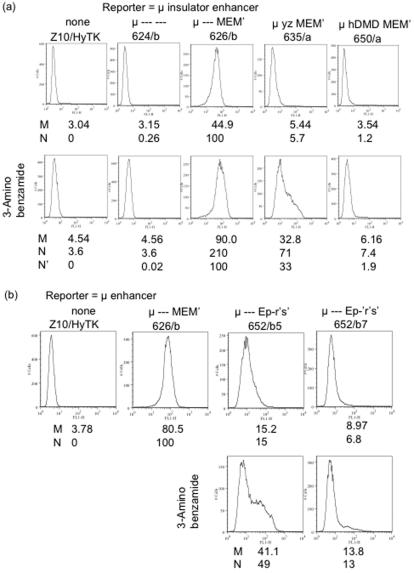
Flow cytometry of cells treated with 3-aminobenzamide. The indicated cells, grown either in normal medium or in medium supplemented with 3-aminobenzamide, were analyzed for intracellular μ protein by flow cytometry, as described in Figure 3. Because 3-aminobenzamide also affected the fluorescence of the μ-negative cells, Z10/HyTK, the normalized fluorescence (N or N′) for cells grown in 3-aminobenzamide was calculated two ways: For cells bearing the reporter gene, N was calculated by subtracting the mean fluorescence of the parental cell line, Z10/HyTK, grown in normal medium; N′ was calculated by subtracting the mean fluorescence for cells grown in 3-aminobenzamide.

In some, if not all, cases the activity of mammalian insulators depends on the protein CTCF. CTCF is a substrate for polyadenylribosylpolymerase (PARP), and treatment of cells with 3-aminobenzamide (3AB), an inhibitor of PARP, increased expression of many CTCF-associated genes [16]. These results suggested that the activity of many insulators might require polyadenylribosylation of CTCF. These considerations suggested that treatment with 3AB might both increase expression and reduce variegation for reporter genes with an insulated enhancer. However, other proteins in chromatin are also substrates for PARP, and an alternative explanation for the 3AB effect was that gene expression is dampened by another (unspecified) PARP-dependent substrate in chromatin. This possibility suggested that 3AB might increase expression and reduce variegation both for reporters with an insulated enhancer and reporters with a mutant enhancer. To test these possibilities, we examined the effects of 3AB on both types of reporters. For comparison we also assayed reporters bearing previously tested insulators, the DMD segments of the mouse (#648) and human (#650) igf2/H19 locus. The activity of these segments was assayed by Northern blot (Fig. 6), flow cytometry (Fig. 7), and ELISA (no shown). For most reporter genes – both those bearing the *gpt*-derived insulator, and the igf2/H19 insulators, as well as those bearing the mutant enhancers – 3AB caused significant (usually ∼3 fold) increases in expression. Flow cytometry indicated that 3AB did not reduce the heterogeneity and even increased heterogeneity greatly in some cases, *viz*., enhancer segment Ep′q′r′ (#652) and the partially insulated enhancer of #635 (Fig. 6). After transfer of the cells back to normal medium, the changes induced by 3AB were reversed (data not shown). The significance of our finding that 3AB increased expression of both insulated and mutant enhancers is considered further in the Discussion.

## Discussion

Our results provide information on several topics:

- Role of the 3′ matrix attachment region;
- Distance and orientation dependence of an enhancer.
- Inhibition of gene expression by the *gpt* cassette;
- Requirements for variegated gene expression.
- Function of the enhancer.

### Role of the 3′ MAR

The function of the matrix attachment regions (MARs) that flank the core enhancer has been elusive. As assayed in transgenic mice, the full enhancer, MEM′, stimulates transcription only in lymphoid cells [17]. Analysis of tissue specificity using transfected cell lines indicated that the core enhancer alone stimulates expression in non-lymphoid cells, but that flanking segments that include the MARs inhibit expression in non-lymphoid cells and in this way render the enhancer activity lymphoid-specific [18]–[20]. In the context of models in which MARs isolate functional chromosomal domains, these results suggested that in non-lymphoid cells the MARs might prevent the enhancer from interacting with the promoter.

The importance of the MARs differs strikingly in transfected cell lines and transgenic mice. As noted above, MARs are not required for high level expression of an immunoglobulin gene that has been transfected into B-cell lines. By contrast, MARs are required for transgene expression in (Abelson) B-cell lines that are made from transgenic mice [12]. It was suggested that the requirement for the MARs in transgenic mice might arise because the transgene becomes methylated in early development, and, in fact, if vector DNA is methylated prior to transfecting the cell line, MARs are required for enhancer-stimulated expression [21]. Further work showed that MARs extend the region of accessible DNA, i.e., DNA that was inaccessible when 1 kb from the core enhancer was accessible when the enhancer was adjoined by the MARs [12], [22].

The difference between transfected cell lines and cell lines derived from transgenic mice suggested that the MARs function only to establish an active locus. However, an ongoing role for the MARs is implied by the discovery of two proteins, Bright and SatB2, that stimulate expression in transfected cell lines, if the reporter gene is linked to the full enhancer (MEM′) but not when it is linked to only the core enhancer [11], [23].

The foregoing analyses in which the MARs stimulated various reporter genes, either in cell lines or in transgenic mice, also contrast with the observation that deleting the intronic MARs from the endogenous IgH locus of ES cells did not affect IgH expression in B-cells [24]. However, this difference between expression of IgH-derived transgenes and the modified IgH locus might simply reflect the contribution of other MARs in the endogenous locus that were absent in the transgenes.

In the present work, we found that the core enhancer, which was active in its normal intronic position, ∼1.4 kb 3′ of the promoter, was inactive when placed ∼6 kb 3′ of the promoter. However, when the core enhancer was adjoined to the 3′ matrix attachment region (MAR), this compound element, EM′, was active in both positions. These results resemble the finding noted above that the MARs extended the region of DNA that is rendered accessible by the core enhancer. That is, the results from both experimental systems suggest that the MARs increase the effective range of the core enhancer. However, the scale is different in the two experimental systems: in the present system the core enhancer stimulated expression from a promoter that was 1.4 kb distal; in the accessibility analysis, DNA that was only 1 kb distal from the core enhancer remained inaccessible. It might be important that the accessibility analysis used cell lines derived from transgenic mice, where the DNA passed through the germ line and was subject to dense methylation. Taken together, these results thus suggest that the core enhancer can be blocked either by a short segment of densely methylated DNA or by a longer, relatively unmethylated segment, i.e., the activity of the core enhancer is distance-dependent. Additional work might be able to establish whether the MARs render the enhancer independent or less dependent on distance.

Our analysis suggests that the M′ segment includes at least two protein binding sites, in that both the 260 bp segment denoted p′ and the 680 bp segment, q′r′s′, are required for the activity provided by M′. Two proteins – Bright and SatB2 – have been identified that stimulate expression from reporter genes that are linked to the MARs of the IgH locus [11], [23]. The binding sites for Bright lie within the segment Ep′ (#653), which was inactive, thus arguing that the potentiator requires another factor in addition to Bright for activity, perhaps SatB2.

In the analyses of Bright and SatB2, MAR DNA stimulated expression only when the reporter was cotransfected with an expression vector for either Bright of SatB2 [11], [23]. This requirement did not apply in our system, perhaps because the hybridoma cell line that we used had higher levels of Bright and/or SatB2 than did the cell lines used in the cotransfection experiments.

### Orientation dependence of the enhancer

It has often been reported that that enhancers function in both orientations. Three quite different reasons might account for such findings: (1) *true orientation-independence* (enhancers might actually function in a way that is intrinsically independent of orientation); (2) *symmetric structure* (enhancers might often contain two symmetrically arranged functional units, i.e., orientation independence is a consequence of the built-in redundancy of these enhancers but not of their basic function); (3) deficient experimental design (reporter genes are usually assayed on a circular DNA molecule or in a tandem array of vectors lying in both orientations, and these circumstances present the enhancer, inadvertently, but nonetheless effectively, in both relative orientations. In our LoxP/Cre system, only a single reporter gene was assayed and the orientation of the enhancer was unambiguous. Our finding that the inverted segment, denoted M′E, in #655, was inactive indicates that the activity of this enhancer, EM′, was orientation-dependent. More work will be needed to establish which component – E , M′ or both – is orientation-dependent and whether the longer segments that were assayed in earlier analyses are constructed with symmetric units.

### Inhibition of reporter gene expression by the gpt cassette

Numerous publications have documented that the selectable markers commonly used to enrich for targeted recombinants sometimes depress the expression of neighboring genes. In particular, our previous work on targeted recombinants of the endogenous IgH locus suggested that the *gpt* cassette is an insulator [15]. The present results show that a segment totally derived from the bacterial *gpt* structural gene was inhibitory, i.e., expression of the μ reporter (and the *neo* gene, Appendix) was depressed in the absence of any SV40-derived regulatory sequence. However, the full *gpt* cassette inhibited more strongly than just the structural gene, and perhaps the SV40 promoter competes for a partially insulated enhancer. A 2.3 kb segment derived from phage λ was inactive (Appendix), thus arguing that prokaryote-derived DNA is not generally inhibitory.

### Requirements for variegated expression

When the reporter gene was under the control of a partially inactivated or partially insulated enhancer, expression of the μ gene was variegated. By contrast, when the reporter gene was linked to an uninsulated, EM′-containing segment (MEM′, PEM′, or EM′), the reporter gene in each of 13 independent cell lines was expressed at a similar high level, and in each case the population of cells was generally homogeneous. We also examined μ expression in 14 independent cell lines in which the reporter genes lacked the q′r′s′ segment (MEP′, PEP′, EP′), and found that these cell lines uniformly failed to express the μ gene at a detectable level. We conclude that variegated expression requires a weakened, but non-zero, enhancer.

Variegated expression in our system had two notable features: (i) clonal heterogeneity, i.e., the mean level of μ expression was different in different clones, and (ii) cellular heterogeneity, i.e., expression of the μ reporter was heterogeneous within a clonal population of cells. Clonal heterogeneity implies that expression can persist in different states through multiple cell divisions. In the present case, these states were metastable, i.e., the mean level of expression was a sufficiently stable property to be inherited by most subclones, but sufficiently unstable that subclones with different average levels of expression were sometimes identified by screening ∼10 subclones.

The variegation seen here resembles the case of targeted recombinants of the endogenous IgH locus, where deletion of the intronic enhancer creates conditions in which the μ gene can persist in very different states of expression that are maintained by a (still undefined) *cis*-acting mechanism [6]–[8]. There are also several differences between variegation in the endogenous and reporter genes. In the endogenous locus, variegated expression ranged from undetectable up to the level for a full enhancer, whereas variegated expression of the reporter gene reached only a fraction of the full level. Also, μ expression in the endogenous locus was variegated in the presence of the full *gpt* cassette, while in the present system, the full *gpt* cassette rendered expression undetectable. These differences between the reporter and endogenous genes might merely reflect the fact that these two genes are in different genomic contexts, linked to different regulatory elements that yield different degrees of variegation. Alternatively, simply screening more clones might have revealed a greater range of reporter expression. However, it might also be important that the endogenous locus, unlike the reporter gene, was expressed at a high level prior to removing the enhancer, i.e., in some way prior high level expression modifies the locus and allows wider ranging variegation.

Why was variegated expression seen only for weakened enhancers? Previous work with a regulatable enhancer revealed that continued, high level expression of an (initially) enhancer-dependent gene can sometimes render expression enhancer-independent [25]. Such a result suggests that the active and inactive states of expression are each autocatalytic: that expression above a specific threshold increases the likelihood that the locus will remain active, while expression below that threshold decreases the likelihood of activity. The importance of weakening the enhancer might be to set expression near the putative threshold.

### Effects of inhibiting PARP

In the case of the imprinted igf2/H19 locus, the different states of the imprinted genes are thought to correspond to different states of the insulator, which in turn are thought to correspond to different patterns of cytidine methylation that allow or disallow binding of the insulator-mediating protein CTCF [13], [14]. This view is further supported by the finding that CTCF is a substrate for PARP, and that inhibition of PARP with 3AB induced expression of the insulated allele [16]. These findings suggested that variegation in the IgH locus might also reflect different states of an insulator. However, this explanation does not account for variegated expression that we found with the mutated enhancers, as these reporter genes did not include an insulator. Nor does it account for our finding that 3AB increased expression from reporters with the mutant enhancers. Rather, these observations suggest the unitary hypothesis that a weak enhancer allows expression to occur at multiple distinct levels, that the role of an insulator is to weaken the enhancer, and that weak expression *per se* can be increased by 3AB, e.g., by the effects of blocking polyadenylribosylation of other components of chromatin [26]. These considerations raise the question whether other methods of weakening expression, such as reducing the strength of the promoter or reducing the concentration of specific transcription factors would likewise allow different states of expression that can be transmitted to progeny cells.

### Function of the enhancer

As measured by the corrected coefficient of variation, weakened enhancers yielded colonies in which expression across the population of cells was more heterogeneous than in the case of the full enhancer. What might be the explanation for this increased cellular heterogeneity? Inasmuch as cells switch from one state to another, clonal heterogeneity must contribute to cellular heterogeneity. However, switching *per se* seems too slow to account fully for cellular heterogeneity. We have considered two additional sources of cellular heterogeneity in μ expression. One source is the variation expected simply because the mean number of RNA molecules per cell was small. However, as calculated in Methods, we estimate that there were on average 20 µ RNA molecules per cell in the variegated clones such as #651 and #654 that produced ∼2% as much μ RNA as #626. The coefficient of variation for a Poisson distribution with a mean of 20 molecules is ∼20%, thus less than 50% and 150% that were found for #651 and #654, respectively (Fig. 5). These calculations therefore argue that the cellular heterogeneity in the variegating clones exceeded the variation expected for a Poisson distribution.

We have also considered an explanation for cellular heterogeneity based on how an enhancer might function. Several different lines of investigation argue that an enhancer is needed continually [27], [28], that it remains close to the promoter [29]–[31], and that it facilitates delivery of RNA polymerase to the promoter [32]. Other analyses of the effects of ablating enhancers have suggested a different model in which (a) the locus can exist in either an expressed or silent state, (b) the rate of transcription is determined by the promoter or other non-enhancer elements, and (c) an enhancer serves to increase the average duration of the expressed state and/or to decrease the average duration of the silent state (reviewed in [5]). This model might account for cellular heterogeneity, if the duration of the silent or expressed state is itself a stochastic feature, akin to a half-life. To illustrate, we consider first the case in which the half-lives of both the expressed and silent states are short, i.e., much less than the cell division time: in this case, cells will typically undergo *many* expressed and silent periods. Because almost all cells will have gone through *many* expressed and silent periods, μ mRNA content will be relatively homogeneous in the population. However, if the half-life of either the expressed or silent state approaches cell division time then cells will typically differ greatly in μ mRNA content. In the context of this model, our finding that cellular heterogeneity is less for cells expressing more μ mRNA suggests that a major effect of the IgH enhancer is to *decrease* the half-life of the silent state. That is, by shortening the half-life of the silent state an enhancer increases the average μ mRNA content, and by shortening the half-life of the silent state an enhancer increases the number of silent and expressed periods and thereby decreases the cellular heterogeneity. Thus, the effect of the enhancer on a single parameter of transcription – the half-life of the silent state – can account for both cellular and clonal heterogeneity.

### Significance

Here we have shown that linking a weakened enhancer to a reporter gene allowed the reporter gene to exist in more than one state of expression. Moreover, that state was persistent, i.e., the particular state was usually transmitted to progeny cells, implying that some form of differentiation had occurred. Although we have related variegation only to *cis*-acting elements – mutations and insulators – our observations suggest that other mechanisms that provide weak enhancer activity, such as limiting the available enhancer-binding proteins, might also result in variegated expression. Variegated expression in general, and monoallelic expression in particular, might require that that activation of expression be a low probability event. It seems reasonable that a weak enhancer or limited access to transcription factors could be a mechanism for activating at low probability. The present case of expression from a weakened enhancer closely resembles the behavior of the IL4 gene, in which many different levels of expression are possible [4]. This similarity suggests that provision of weak enhancer activity might also be a normal mechanism for generating differentiated populations in the course of development.

## Materials and Methods

### Vectors and DNA segments

The Cre expression vector and LoxP vector were obtained from E. Bouhassira[10]. The structures of the replacement vectors are described in the text and in Figure 1; the nucleotide sequences of the replacement and target vectors are given in the additional files of [9].

The DMD insulator segment used in the RMCE vector was derived by PCR from the mouse igf2/H19 DMD (differentially methylated domain) and corresponded to nucleotides 1540-3173 of the Genbank sequence MMU19619 [5′-TGGACTCGGACTCCC-//-GGGTCCGCTGTGACA-3′].

### Recombination-mediated cassette exchange

We have previously described the construction of the recipient cell line, Z10/HyTK, transfection, selection of gancyclovir-resistant cells, and the methods for identifying transfectants in which a complete and intact reporter cassette has replaced the target cassette in one particular orientation[9].

The original names of transfectants were simplified for ease of presentation: 613/1.19.1 = a, /4.7.3 = b; 616/A1.6.5 = a, /A2.14.5 = b; 624//C2.10.1 = a, B.10.1 = b; 625/ C1.7.1 = a, /A1.3.1 = b; 626/B1.1.1 = a, /B2.16.1 = b; 629/1.9.1 = a, /A1.6.1 = b; 630/2.2.1 = a, /A2.4.2 = b; 635/C2.23 = a, /A1.2.2 = b; 637/B2.4.2 = a, /A1.2.1 = b; 639/A2.15.1 = a, /B1.9.1 = b; 640/C.5.2 = a, /E2.12.1 = b; 642/20.88 = a, /10.55 = b; 643/A1.1.1 = a, /C1.14.1 = b; 644/B1.4.1 = a, /C12.1 = b; 651/A122.7 = a, /A183.8 = b; 652/B1.22.4 = a, /A1.9.3 = b; 653/A1.12.1 = a, /A2.2.1 = b; 654/C2.5.1 = a, /3.2.1 = b; 655/2.13.2 = a, /1A.15.1 = b

### Analysis of μ expression

ELISA's and Northern blots were done by standard methods. Flow cytometry of intracellular IgM has been described [6].

### Cell culture

The materials for cell culture have been described [33]. As indicated in the text, cells were treated with 6.7 mM 3-aminobenzamide for approximately seven days prior to analysis of μ RNA and protein.

### Calculation of μ RNA/cell

Other analyses have estimated that large cells, such as our polyploid hybridoma cells, contain approximately 400 pg protein and 30 pg RNA per cell, and that mRNA constitutes 3% of the total, or ∼1 pg/cell [34]–[36]. Assuming that the typical mRNA is ∼2 kb, 1 pg mRNA/cell corresponds to 10^6^ molecules/cell. Our ELISA measurements indicated that a culture of the Sp6 hybridoma, initiated at a concentration of 3×10^5^ cells/ml and growing with a doubling time of ∼16 hr, secreted 3.5 µg/ml IgM (2.5 µg/ml μ chain (∼576 amino acids long)) and produced∼4.5×10^5^ cells/ml in ∼20 hr. These cells therefore made 400 pg protein/cell × 4.5×10^5^ cells/ml  =  180 µg/ml total protein/ml. The μ chain thus corresponds to 2.5/180  =  ∼1.5% of the total protein. Assuming that mRNA length is proportional to protein length, and that the average protein is ∼450 amino acids long, the number of μ mRNA molecules in Sp6 would be 10^6^ × (0.015) × 450/576  =  ∼1×10^4^. The cells expressing reporter #626 contained ∼11% as much μ RNA as Sp6, implying that these cells each contained on average ∼1100 molecules of μ RNA.

## Supporting Information

Figure S1 Insulator activity of gpt
(0.65 MB PDF)
