# Supplementary material for: A Weakened Transcriptional Enhancer Yields Variegated Gene Expression
Source: PLoS One. 2006 Dec 20;1(1):e33. doi: 10.1371/journal.pone.0000033 (PMC1762374; doi:10.1371/journal.pone.0000033)
Supplement: Figure S1 — Insulator activity of gpt (0.65 MB PDF) [file pone.0000033.s001.pdf]

Relative Enhancer Function

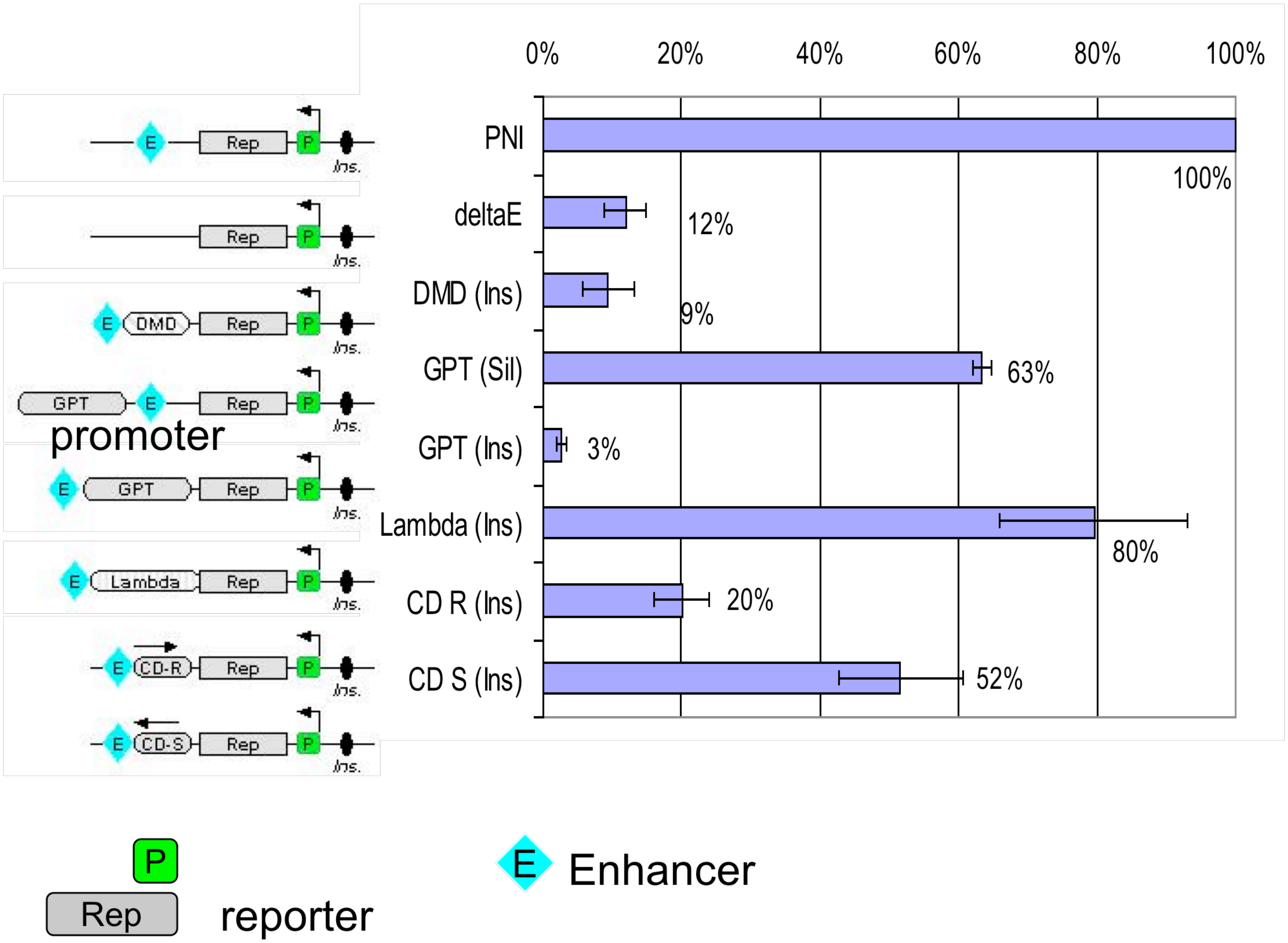

Vectors

PNI – vector with enhancer and without insulator or silencer; all values are relative to PNI.
deltaE – enhancerless vector
DMD (Ins) – Differentially Methylated Domain from the Igf2/H19 locus in the insulator position (positive control for insulator function).
GPT(Ins) – full length GPT cassette in the insulator position
Lambda (Ins) – Lambda HindIII 2.3Kb fragment in the insulator position (control for distance-specific effects).
CD-R (ins) – segment of the GPT cassette in the insulator position with reverse orientation with respect to the promoter.
CD-S (Ins) - segment of the GPT cassette in the insulator position with same orientation with respect the promoter.
GPT (Sil) – full length GPT cassette in the "silencer" position.

Figure S1: Insulator Activity of *gpt* Cassette

Analysis. Bell, et al. (13) developed a simple assay for the insulator activity using vectors that bear a neo (G418 resistance) gene in which expression depends on an enhancer. Insertion of DNA segments with insulator activity between the promoter for the G418 gene and the enhancer decreases the frequency of G418-resistant transfectants. The structure of the vectors and their capacity to generated G418-resistant transfectants are presented in the figure. The results indicate that the complete *gpt* cassette decreased the frequency of G418-resistance by ~30 fold in the insulator site but only 1.5 fold in the silencer site. In the insulator site the *gpt* cassette decreased G418 resistance below the value for the enhancer-deficient vector. Taken together, these results suggest that the *gpt* cassette has two activities: a slight silencer activity and a major insulator activity. Most of this insulator activity is present in the segment in the CD R and CD S vectors, a segment which is derived entirely from the E. coli *gpt* structural gene. By comparison the segment derived from phage lambda had little, if any, insulator activity.

Construction of vectors and assay for insulator activity. Vectors PNI and DMD were obtained from Dr. G. Felsenfeld (1). The ΔE vector was generated by digestion of PNI with EcoRI and religation, thereby releasing the 1.2Kb enhancer fragment from the PNI backbone. The GPT (Ins) and GPT (Sil) vectors bearing the full-length GPT cassette and CD R and CD S bearing the 1.1Kb common deletion fragment from the *gpt* gene were generated by PCR from the ΔMEMS vector (20). The primers were designed to add an AscI linker. The PCR products (bearing the AscI site) were cloned directly into the AscI (insulator) site in PNI or blunt-end cloned into the blunted-NdeI (silencer) site. Same and reversed orientations are with regard to the direction of transcription of the *neo* and *gpt* cassettes. To generate the λ (Ins) vector, the 2.3Kb fragment from the λ HindIII (Fermentas) was isolated by gel purification and blunt-end ligated into a blunted AscI site.

K562 cells (obtained from Dr. F. Tsui), were maintained in αMEM supplemented with 10% fetal bovine serum. Cells were transfected by electroporation. For electroporation, cells were grown to a density of $3x10^5$ – $5x10^5$ cells/mL in a 250 mL flask. Vector DNA was linearized with SalI, purified with Qiagen PCR clean kit, and resuspended in H_2O. 1ug of SalI linearized vector DNA in PBS was used for each transformation. Cells were pulsed once in a 0.4cm cuvette in a BioRad gene pulser at 1kV and 25μF. Electroporated cells were resuspended in αMEM supplemented with 10% fetal bovine serum. Three days post electroporation, cells were selected in 0.9mg/mL of G418 and plated into a 96 well plate in duplicate at $2x10^3$ and $1x10^4$cells/well. 21 days post selection, growth-positive wells were scored and the frequency of G418-resistant cells was calculated according to the Poisson distribution. The results presented are the average of three separate experiments.

Primers:

Full length GPT:

5'-GGCGCGCCAGAAGAAAAAGAGAAGCAAGGGGG

5'-GGCGCGCCAGAAGACAGGGGAGAAGGGCCTTAA;

CD R and CD S:

5'GGCGCGCCGACGCGGACTCATGTGAAATACTGG

5'GGCGCGCCCACTGCTCCCATTCATCAGTTCCAT
